# Supplementary material for: Factors Affecting Enteric Emission Methane and Predictive Models for Dairy Cows
Source: Animals (Basel). 2023 Jun 2;13(11):1857. doi: 10.3390/ani13111857 (PMC10252078; doi:10.3390/ani13111857)
Supplement: Supplementary file 1 [file animals-13-01857-s001.zip › animals-2403067-supplementary.pdf]

animals

Article

Factors Affecting Methane Enteric Emission and Predictive Models for Dairy Cows

Andrea Beltrani Donadia[1], Rodrigo Nazaré Santos Torres[1], Henrique Melo da Silva[1], Suziane Rodrigues Soares[1], Aaron Kinyu Hoshide[2,3], and André Soares de Oliveira[1,*]

[1] Dairy Cattle Research Laboratory, Universidade Federal de Mato Grosso, Campus Sinop, Sinop, Mato Grosso, Brazil, 78555-267, Brazil.
[2] College of Natural Sciences, Forestry, and Agriculture, The University of Maine, Orono, Maine, 04469-5782, USA.
[3] AgriSciences, Universidade Federal de Mato Grosso, Campus Sinop, Sinop, Mato Grosso, Brazil, 78555-267, Brazil.
* Correspondence: andre.oliveira@ufmt.br; Tel.: +55-66-99942-3733.

Supplemental Materials

animals
MDPI

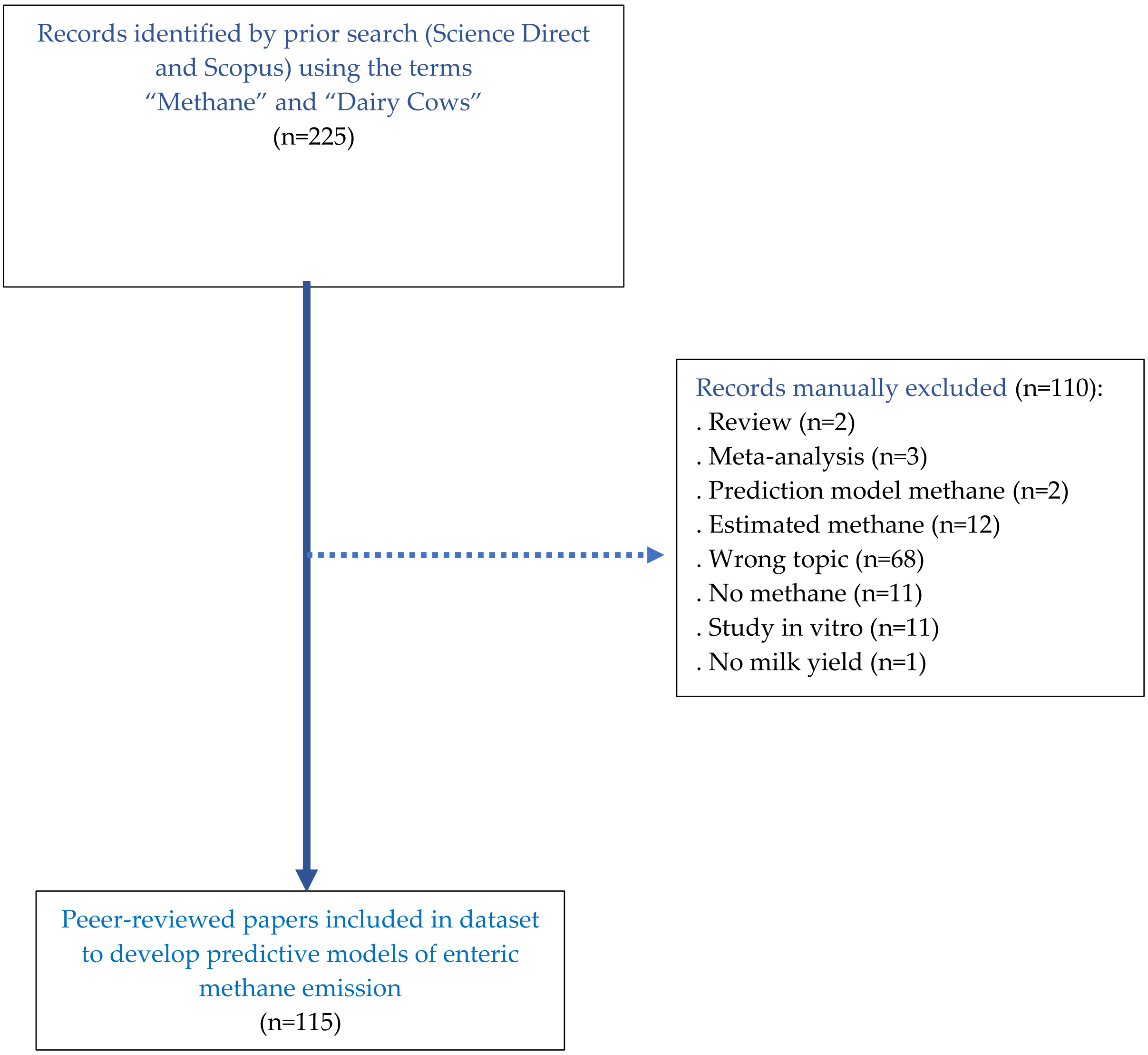

Figure S1. Flowchart showing exclusion/inclusion criteria for selection of peer-reviewed papers used included in dataset to develop predictive models of enteric methane emission in dairy cows.

Agle, M.; Hristov, A.N.; Zaman, S.; Schneider, C.; Ndegwa, P.M.; Vaddella, V.K. Effect of dietary concentrate on rumen fermentation, digestibility, and nitrogen losses in dairy cows. *J. Dairy Sci.* **2010**, *93(9)*, 4211–4222. https://doi.org/10.3168/jds.2009-2977.

Aguerre, M.J.; Wattiaux, M.A.; Powell, J.M.; Broderick, G.A.; Arndt, C. Effect of forage-to-concentrate ratio in dairy cow diets on emission of methane, carbon dioxide, and ammonia, lactation performance, and manure excretion. *J. Dairy Sci.* **2011**, *94(6)*, 3081–3093. https://doi.org/10.3168/jds.2010-4011.

Alstrup, L.; Hellwing, A.L.F.; Lund, P.; Weisbjerg, M.R. Effect of fat supplementation and stage of lactation on methane production in dairy cows. *Anim. Feed Sci. Technol.* **2015**, *207*, 10–19. https://doi.org/10.1016/j.anifeedsci.2015.05.017.

Beauchemin, K.A.; McGinn, S.M.; Benchaar, C.; Holtshausen, L. Crushed sunflower, flax, or canola seeds in lactating dairy cow diets: Effects on methane production, rumen fermentation, and milk production. *J. Dairy Sci.* **2009**, *92(5)*, 2118–2127. https://doi.org/10.3168/jds.2008-1903.

Benchaar, C. Diet supplementation with cinnamon oil, cinnamaldehyde, or monensin does not reduce enteric methane production of dairy cows. *Animal* **2015**, *10(3)*, 418–425. https://doi.org/10.1017/S175173111500230X.

Benchaar, C.; Hassanat, F.; Gervais, R.; Chouinard, P.Y.; Julien, C.; Petit, H.V.; Massé, D.I. Effects of increasing amounts of corn dried distillers grains with solubles in dairy cow diets on methane production, ruminal fermentation, digestion, N balance, and milk production. *J. Dairy Sci.* **2013**, *96(4)*, 2413–2427. https://doi.org/10.3168/jds.2012-6037.

Benchaar, C.; Hassanat, F.; Petit, H.V. Dose-response to eugenol supplementation to dairy cow diets: Methane production, N excretion, ruminal fermentation, nutrient digestibility, milk production, and milk fatty acid profile. *Anim. Feed Sci. Technol.* **2015**, *209*, 51–59. https://doi.org/10.1016/j.anifeedsci.2015.07.027.

Branco, A.F.; Giallongo, F.; Frederick, T.; Weeks, H.; Oh, J.; Hristov, A.N. Erratum to "Effect of technical cashew nut shell liquid on rumen methane emission and lactation performance of dairy cows" (J. Dairy Sci. 98:4030-4040). *J. Dairy Sci.* **2015**, *98(7)*, 5018. https://doi.org/10.3168/jds.2015-98-7-5018.

Brask, M.; Lund, P.; Hellwing, A.L.F.; Poulsen, M.; Weisbjerg, M.R. Enteric methane production, digestibility and rumen fermentation in dairy cows fed different forages with and without rapeseed fat supplementation. *Anim. Feed Sci. Technol.* **2013**, *184*, 67–79. https://doi.org/10.1016/j.anifeedsci.2013.06.006.

Cavanagh, A.; McNaughton, L.; Clark, H.; Greaves, C.; Gowan, J.M.; Pinares-Patino, C.; Dalley, D.; Vlaming, B.; Molano, G. Methane emissions from grazing Jersey x Friesian dairy cows in mid lactation. *Aust. J. Exp. Agric.* **2008**, *48(2)*, 230–233. https://doi.org/10.1071/EA07277.

Chung, Y.H.; Zhou, M.; Holtshausen, L.; Alexander, T.W.; McAllister, T.A.; Guan, L.L.; Oba, M.; Beauchemin, K.A. A fibrolytic enzyme additive for lactating Holstein cow diets: Ruminal fermentation, rumen microbial populations, and enteric methane emissions. *J. Dairy Sci.* **2012**, *95(3)*, 1419–1427. https://doi.org/10.3168/jds.2011-4552.

Dall-Orsoletta, A.C.; Almeida, J.G.R.; Carvalho, P.C.F.; Savian, J.V.; Ribeiro-Filho, H.M.N. Ryegrass pasture combined with partial total mixed ration reduces enteric methane emissions and maintains the performance of dairy cows during mid to late lactation. *J. Dairy Sci.* **2016**, *99(6)*, 4374–4383. https://doi.org/10.3168/jds.2015-10396.

Doreau, M.; Ferlay, A.; Rochette, Y.; Martin, C. Effects of dehydrated lucerne and soya bean meal on milk production and composition, nutrient digestion, and methane and nitrogen losses in dairy cows receiving two different forages. *Animal* **2014**, *8(3)*, 420–430. https://doi.org/10.1017/S1751731113002206.

Dorich, C.D.; Varner, R.K.; Pereira, A.B.D.; Martineau, R.; Soder, K.J.; Brito, A.F. Short communication: Use of a portable, automated, open-circuit gas quantification system and the sulfur hexafluoride tracer technique for measuring enteric methane emissions in Holstein cows fed ad libitum or restricted. *J. Dairy Sci.* **2015**, *98(4)*, 2676–2681. https://doi.org/10.3168/jds.2014-8348.

Enriquez-Hidalgo, D.; Gilliland, T.; Deighton, M.H.; O'Donovan, M.; Hennessy, D. Milk production and enteric methane emissions by dairy cows grazing fertilized perennial ryegrass pasture with or without inclusion of white clover. *J. Dairy Sci.* **2014**, *97(3)*, 1400–1412. https://doi.org/10.3168/jds.2013-7034.

Foley, P.A.; Kenny, D.A.; Lovett, D.K.; Callan, J.J.; Boland, T.M.; O'Mara, F.P. Effect of DL-malic acid supplementation on feed intake, methane emissions, and performance of lactating dairy cows at

pasture. *J. Dairy Sci.* **2009**, *92(7)*, 3258–3264. https://doi.org/10.3168/jds.2008-1633.

Foth, A.J.; Brown-Brandl, T.; Hanford, K.J.; Miller, P.S.; Garcia Gomez, G.; Kononoff, P.J. Energy content of reduced-fat dried distillers grains with solubles for lactating dairy cows. *J. Dairy Sci.* **2015**, *98(10)*, 7142–7152. https://doi.org/10.3168/jds.2014-9226.

Garg, M.R.; Sherasia, P.L.; Bhanderi, B.M.; Phondba, B.T.; Shelke, S.K.; Makkar, H.P.S. Effects of feeding nutritionally balanced rations on animal productivity, feed conversion efficiency, feed nitrogen use efficiency, rumen microbial protein supply, parasitic load, immunity and enteric methane emissions of milking animals under field conditions. *Anim. Feed Sci. Technol.* **2013**, *179*, 24–35. https://doi.org/10.1016/j.anifeedsci.2012.11.005.

Gidlund, H.; Hetta, M.; Krizsan, S.J.; Lemosquet, S.; Huhtanen, P. Effects of soybean meal or canola meal on milk production and methane emissions in lactating dairy cows fed grass silage-based diets. *J. Dairy Sci.* **2015**, *98(11)*, 8093–8106. https://doi.org/10.3168/jds.2015-9757.

Grainger, C.; Auldist, M.J.; Clarke, T.; Beauchemin, K.A.; McGinn, S.M.; Hannah, M.C.; Eckard, R.J.; Lowe, L.B. Use of monensin controlled-release capsules to reduce methane emissions and improve milk production of dairy cows offered pasture supplemented with grain. *J. Dairy Sci.* **2008**, *91(3)*, 1159–1165. https://doi.org/10.3168/jds.2007-0319.

Grainger, C.; Clarke, T.; Auldist, M.J.; Beauchemin, K.A.; McGinn, S.M.; Waghorn, G.C.; Eckard, R.J. Potential use of Acacia mearnsii condensed tannins to reduce methane emissions and nitrogen excretion from grazing dairy cows. *Can. J. Anim. Sci.* **2009**, *89(2)*, 241–251. https://doi.org/10.4141/cjas08110.

Grainger, C.; Clarke, T.; McGinn, S.M.; Auldist, M.J.; Beauchemin, K.A.; Hannah, M.C.; Waghorn, G.C.; Clark, H.; Eckard, R.J. Methane emissions from dairy cows measured using the sulfur hexafluoride (SF6) tracer and chamber techniques. *J. Dairy Sci.* **2007**, *90(6)*, 2755–2766. https://doi.org/10.3168/jds.2006-697.

Grainger, C.; Williams, R.; Clarke, T.; Wright, A.D.G.; Eckard, R.J. Supplementation with whole cottonseed causes long-term reduction of methane emissions from lactating dairy cows offered a forage and cereal grain diet. *J. Dairy Sci.* **2010**., *93(6)*, 2612–2619. https://doi.org/10.3168/jds.2009-2888.

Grainger, C.; Williams, R.; Eckard, R.J.; Hannah, M.C. A high dose of monensin does not reduce methane emissions of dairy cows offered pasture supplemented with grain. *J. Dairy Sci.* **2010**, *93(11)*, 5300–5308. https://doi.org/10.3168/jds.2010-3154.

Guyader, J.; Doreau, M.; Morgavi, D.P.; Gérard, C.; Loncke, C.; Martin, C. Long-term effect of linseed plus nitrate fed to dairy cows on enteric methane emission and nitrate and nitrite residuals in milk. *Animal* **2016**, *10(7)*, 1173–1181. https://doi.org/10.1017/S1751731115002852.

Guyader, J.; Eugène, M.; Doreau, M.; Morgavi, D.P.; Gérard, C.; Martin, C. Tea saponin reduced methanogenesis in vitro but increased methane yield in lactating dairy cows. *J. Dairy Sci.* **2017**, *100(3)*, 1845–1855. https://doi.org/10.3168/jds.2016-11644.

Haisan, J.; Sun, Y.; Guan, L.L.; Beauchemin, K.A.; Iwaasa, A.; Duval, S.; Barreda, D.R.; Oba, M. The effects of feeding 3-nitrooxypropanol on methane emissions and productivity of Holstein cows in mid lactation. *J. Dairy Sci.* **2014**, *97(5)*, 3110–3119. https://doi.org/10.3168/jds.2013-7834.

Hammond, K.J.; Jones, A.K.; Humphries, D.J.; Crompton, L.A.; Reynolds, C.K. Effects of diet forage source and neutral detergent fiber content on milk production of dairy cattle and methane emissions determined using GreenFeed and respiration chamber techniques. *J. Dairy Sci.* **2016**, *99(10)*, 7904–7917. https://doi.org/10.3168/jds.2015-10759.

Haque, M.N.; Cornou, C.; Madsen, J. Estimation of methane emission using the CO2 method from dairy cows fed concentrate with different carbohydrate compositions in automatic milking system. *Livest. Sci.* **2014**, *164*, 57–66. https://doi.org/10.1016/j.livsci.2014.03.004.

Hart, K.J.; Huntington, J.A.; Wilkinson, R.G.; Bartram, C.G.; Sinclair, L.A. The influence of grass silage-to-maize silage ratio and concentrate composition on methane emissions, performance and milk composition of dairy cows. *Animal* **2014**, *9(6)*, 983–991. https://doi.org/10.1017/S1751731115000208.

Hassanat, F.; Gervais, R.; Massé, D.I.; Petit, H.V.; Benchaar, C. Methane production, nutrient digestion, ruminal fermentation, N balance, and milk production of cows fed timothy silage- or alfalfa silage-based diets. *J. Dairy Sci.* **2014**, *97(10)*, 6463–6474. https://doi.org/10.3168/jds.2014-8069.

Hatew, B.; Bannink, A.; van Laar, H.; de Jonge, L.H.; Dijkstra, J. Increasing harvest maturity of whole-plant corn silage reduces methane emission of lactating dairy cows. *J. Dairy Sci.* **2016**, *99(1)*, 354–368. https://doi.org/10.3168/jds.2015-10047.

Hatew, B.; Cone, J.W.; Pellikaan, W.F.; Podesta, S.C.; Bannink, A.; Hendriks, W.H.; Dijkstra, J. Relationship

between in vitro and in vivo methane production measured simultaneously with different dietary starch sources and starch levels in dairy cattle. *Anim. Feed Sci. Technol.* **2015**, *202*, 20–31. https://doi.org/10.1016/j.anifeedsci.2015.01.012.

Hellwing, A.L.F.; Weisbjerg, M.R.; Møller, H.B. Enteric and manure-derived methane emissions and biogas yield of slurry from dairy cows fed grass silage or maize silage with and without supplementation of rapeseed. *Livest. Sci.* **2014**, *165*, 189–199. https://doi.org/10.1016/j.livsci.2014.04.011.

Holter, J.B.; Colovos, N.F.; Clark, R.M.; Koes, R.M.; Davis, H.A.; Urban, W.E. Urea for Lactating Dairy Cattle. V. Concentrate Fiber and Urea in a Corn Silage-High Concentrate Ration. *J. Dairy Sci.* **1971**, *54(10)*, 1475–1481. https://doi.org/10.3168/jds.S0022-0302(71)86050-2.

Hristov, A.N.; Lee, C.; Cassidy, T.; Long, M.; Heyler, K.; Corl, B.; Forster, R. Effects of lauric and myristic acids on ruminal fermentation, production, and milk fatty acid composition in lactating dairy cows. *J. Dairy Sci.* **2011**, *94(1)*, 382–395. https://doi.org/10.3168/jds.2010-3508.

Hristov, A.N.; Oh, J.; Giallongo, F.; Frederick, T.W.; Harper, M.T.; Weeks, H.L.; Branco, A.F.; Moate, P.J.; Deighton, M.H.; Williams, S.R.O.; Kindermann, M.; Duval, S. An inhibitor persistently decreased enteric methane emission from dairy cows with no negative effect on milk production. *Proc. Natl. Acad. Sci. U.S.A.* **2015**, *112(34)*, 10663–10668. https://doi.org/10.1073/pnas.1504124112.

Hristov, A.N.; Varga, G.; Cassidy, T.; Long, M.; Heyler, K.; Karnati, S.K.R.; Corl, B.; Hovde, C.J.; Yoon, I. Effect of Saccharomyces cerevisiae fermentation product on ruminal fermentation and nutrient utilization in dairy cows. *J. Dairy Sci.* **2010**, *93(2)*, 682–692. https://doi.org/10.3168/jds.2009-2379.

Huhtanen, P.; Cabezas-Garcia, E.H.; Utsumi, S.; Zimmerman, S. Comparison of methods to determine methane emissions from dairy cows in farm conditions. *J. Dairy Sci.* **2015**, *98(5)*, 3394–3409. https://doi.org/10.3168/jds.2014-9118.

Huyen, N.T.; Desrues, O.; Alferink, S.J.J.; Zandstra, T.; Verstegen, M.W.A.; Hendriks, W.H.; Pellikaan, W.F. Inclusion of sainfoin (Onobrychis viciifolia) silage in dairy cow rations affects nutrient digestibility, nitrogen utilization, energy balance, and methane emissions. *J. Dairy Sci.* **2016**, *99(5)*, 3566–3577. https://doi.org/10.3168/jds.2015-10583.

Hynes, D.N.; Stergiadis, S.; Gordon, A.; Yan, T. Effects of concentrate crude protein content on nutrient digestibility, energy utilization, and methane emissions in lactating dairy cows fed fresh-cut perennial grass. *J. Dairy Sci.* **2016**, *99(11)*, 8858–8866. https://doi.org/10.3168/jds.2016-11509.

Johansen, M.; Hellwing, A.L.F.; Lund, P.; Weisbjerg, M.R. Metabolisable protein supply to lactating dairy cows increased with increasing dry matter concentration in grass-clover silage. *Anim. Feed Sci. Technol.* **2017**, *227*, 95–106. https://doi.org/10.1016/j.anifeedsci.2017.02.018.

Jonker, A.; Molano, G.; Koolaard, J.; Muetzel, S. Methane emissions from lactating and non-lactating dairy cows and growing cattle fed fresh pasture. *Anim. Prod. Sci.* **2017**, *57(4)*, 643–648. https://doi.org/10.1071/AN15656.

Jonker, A.; Scobie, D.; Dynes, R.; Edwards, G.; De Klein, C.; Hague, H.; McAuliffe, R.; Taylor, A.; Knight, T.; Waghorn, G. Feeding diets with fodder beet decreased methane emissions from dry and lactating dairy cows in grazing systems. *Anim. Prod. Sci.* **2017**, *57(7)*, 1445–1450. https://doi.org/10.1071/AN16441.

Kinsman, R.; Sauer, F.D.; Jackson, H.A.; Wolynetz, M.S. Methane and Carbon Dioxide Emissions from Dairy Cows in Full Lactation Monitored over a Six-Month Period. *J. Dairy Sci.* **1995**, *78*, 2760–2766. https://doi.org/10.3168/jds.S0022-0302(95)76907-7.

Klop, G.; Dijkstra, J.; Dieho, K.; Hendriks, W.H.; Bannink, A. Enteric methane production in lactating dairy cows with continuous feeding of essential oils or rotational feeding of essential oils and lauric acid. *J. Dairy Sci.* **2017**, *100(5)*, 3563–3575. https://doi.org/10.3168/jds.2016-12033.

Klop, G.; Hatew, B.; Bannink, A.; Dijkstra, J. Feeding nitrate and docosahexaenoic acid affects enteric methane production and milk fatty acid composition in lactating dairy cows. *J. Dairy Sci.* **2016**, *99(2)*, 1161–1172. https://doi.org/10.3168/jds.2015-10214.

Lejonklev, J.; Kidmose, U.; Jensen, S.; Petersen, M.A.; Helwing, A.L.F.; Mortensen, G.; Weisbjerg, M.R.; Larsen, M.K. Short communication: Effect of oregano and caraway essential oils on the production and flavor of cow milk. *J. Dairy Sci.* **2016**, *99(10)*, 7898–7903. https://doi.org/10.3168/jds.2016-10910.

Lettat, A.; Hassanat, F., Benchaar, C. Corn silage in dairy cow diets to reduce ruminal methanogenesis: Effects on the rumen metabolically active microbial communities. *J. Dairy Sci.* **2013**, *96(8)*, 5237–5248. https://doi.org/10.3168/jds.2012-6481.

Lopes, J.C.; de Matos, L.F.; Harper, M.T.; Giallongo, F.; Oh, J.; Gruen, D.; Ono, S.; Kindermann, M.; Duval, S.; Hristov, A.N. Effect of 3-nitrooxypropanol on methane and hydrogen emissions, methane isotopic signature, and ruminal fermentation in dairy cows. *J. Dairy Sci.* **2016**, *99(7)*, 5335–5344.

https://doi.org/10.3168/jds.2015-10832.

Lopes, J.C.; Harper, M.T.; Giallongo, F.; Oh, J.; Smith, L.; Ortega-Perez, A.M.; Harper, S.A.; Melgar, A.; Kniffen, D.M.; Fabin, R.A.; Hristov, A.N. Effect of high-oleic-acid soybeans on production performance, milk fatty acid composition, and enteric methane emission in dairy cows. *J. Dairy Sci.* **2017**, *100(2)*, 1122–1135. https://doi.org/10.3168/jds.2016-11911.

Machado, F.S.; Tomich, T.R.; Ferreira, A.L.; Cavalcanti, L.F.L.; Campos, M.M.; Paiva, C.A.V.; Ribas, M.N.; Pereira, L.G.R. Technical note: A facility for respiration measurements in cattle. *J. Dairy Sci.* **2016**, *99(6)*, 4899–4906. https://doi.org/10.3168/jds.2015-10298.

Macome, F.M.; Pellikaan, W.F.; Schonewille, J.T.; Bannink, A.; Van Laar, H.; Hendriks, W.H.; Warner, D.; Cone, J.W. In vitro rumen gas and methane production of grass silages differing in plant maturity and nitrogen fertilisation, compared to in vivo enteric methane production. *Anim. Feed Sci. Technol.* **2017**, *230*, 96–102. https://doi.org/10.1016/j.anifeedsci.2017.04.005.

Martin, C.; Ferlay, A.; Mosoni, P.; Rochette, Y.; Chilliard, Y.; Doreau, M. Increasing linseed supply in dairy cow diets based on hay or corn silage: Effect on enteric methane emission, rumen microbial fermentation, and digestion. *J. Dairy Sci.* **2016**, *99(5)*, 3445–3456. https://doi.org/10.3168/jds.2015-10110.

Martin, C.; Rouel, J.; Jouany, J.P.; Doreau, M.; Chilliard, Y. Methane output and diet digestibility in response to feeding dairy cows crude linseed, extruded linseed, or linseed oil. *J. Anim. Sci.* **2008**, *86(10)*, 2642–2650. https://doi.org/10.2527/jas.2007-0774.

Mata e Silva, B.C.; Lopes, F.C.F.; Pereira, L.G.R.; Tomich, T.R.; Morenz, M.J.F.; Martins, C.E.; Gomide, C.A.M.; Paciullo, D.S.C.; Maurício, R.M.; Chaves, A.V. Effect of sunflower oil supplementation on methane emissions of dairy cows grazing Urochloa brizantha cv. marandu1. *Anim. Prod. Sci.* **2017**, *57(7)*, 1431–1436. https://doi.org/10.1071/AN16470.

Moate, P.J.; Williams, S.R.O.; Grainger, C.; Hannah, M.C.; Ponnampalam, E.N.; Eckard, R.J. Influence of cold-pressed canola, brewers grains and hominy meal as dietary supplements suitable for reducing enteric methane emissions from lactating dairy cows. *Anim. Feed Sci. Technol.* **2011**, *166–167*, 254–264. https://doi.org/10.1016/j.anifeedsci.2011.04.069.

Moate, P.J.; Williams, S.R.O.; Hannah, M.C.; Eckard, R.J.; Auldist, M.J.; Ribaux, B.E.; Jacobs, J.L.; Wales, W.J. Effects of feeding algal meal high in docosahexaenoic acid on feed intake, milk production, and methane emissions in dairy cows. *J. Dairy Sci.* **2013**, *96(5)*, 3177–3188. https://doi.org/10.3168/jds.2012-6168.

Muñoz, C.; Letelier, P.A.; Ungerfeld, E.M.; Morales, J.M.; Hube, S.; Pérez-Prieto, L.A. Effects of pregrazing herbage mass in late spring on enteric methane emissions, dry matter intake, and milk production of dairy cows. *J. Dairy Sci.* **2016**, *99(10)*, 7945–7955. https://doi.org/10.3168/jds.2016-10919.

Muñoz, C.; Wills, D.A.; Yan, T. Effects of dietary active dried yeast (Saccharomyces cerevisiae) supply at two levels of concentrate on energy and nitrogen utilisation and methane emissions of lactating dairy cows. *Anim. Prod. Sci.* **2017**, *57(4)*, 656–664. https://doi.org/10.1071/AN15356.

Muñoz, C.; Yan, T.; Wills, D.A.; Murray, S.; Gordon, A.W. Comparison of the sulfur hexafluoride tracer and respiration chamber techniques for estimating methane emissions and correction for rectum methane output from dairy cows. *J. Dairy Sci.* **2012**, *95(6)*, 3139–3148. https://doi.org/10.3168/jds.2011-4298.

Niu, M.; Appuhamy, J.A.D.R.N.; Leytem, A.B.; Dungan, R.S.; Kebreab, E. Effect of dietary crude protein and forage contents on enteric methane emissions and nitrogen excretion from dairy cows simultaneously. *Anim. Prod. Sci.* **2016**, *56(3)*, 312–321. https://doi.org/10.1071/AN15498.

O'Neill, B.F.; Deighton, M.H.; O'Loughlin, B.M.; Mulligan, F.J.; Boland, T.M.; O'Donovan, M.; Lewis, E. Effects of a perennial ryegrass diet or total mixed ration diet offered to spring-calving Holstein-Friesian dairy cows on methane emissions, dry matter intake, and milk production. *J. Dairy Sci.* **2011**, *94(4)*, 1941–1951. https://doi.org/10.3168/jds.2010-3361.

Odongo, N.E.; Or-Rashid, M.M.; Kebreab, E.; France, J.; McBride, B.W. Effect of supplementing myristic acid in dairy cow rations on ruminal methanogenesis and fatty acid profile in milk. *J. Dairy Sci.* **2007**, *90(4)*, 1851–1858. https://doi.org/10.3168/jds.2006-541.

Olijhoek, D.W.; Hellwing, A.L.F.; Brask, M.; Weisbjerg, M.R.; Højberg, O.; Larsen, M.K.; Dijkstra, J.; Erlandsen, E.J.; Lund, P. Effect of dietary nitrate level on enteric methane production, hydrogen emission, rumen fermentation, and nutrient digestibility in dairy cows. *J. Dairy Sci.* **2016**, *99(8)*, 6191–6205. https://doi.org/10.3168/jds.2015-10691.

Philippeau, C.; Lettat, A.; Martin, C.; Silberberg, M.; Morgavi, D.P.; Ferlay, A.; Berger, C.; Nozière, P. Effects of bacterial direct-fed microbials on ruminal characteristics, methane emission, and milk fatty acid composition in cows fed high- or low-starch diets. *J. Dairy Sci.* **2017**, *100(4)*, 2637–2650.

animals MDPI

https://doi.org/10.3168/jds.2016-11663.
Pirondini, M.; Colombini, S.; Mele, M.; Malagutti, L.; Rapetti, L.; Galassi, G.; Crovetto, G.M. Effect of dietary starch concentration and fish oil supplementation on milk yield and composition, diet digestibility, and methane emissions in lactating dairy cows. *J. Dairy Sci.* **2015**, *98(1)*, 357–372. https://doi.org/10.3168/jds.2014-8092.
Reynolds, C.K.; Humphries, D.J.; Kirton, P.; Kindermann, M.; Duval, S.; Steinberg, W. Effects of 3-nitrooxypropanol on methane emission, digestion, and energy and nitrogen balance of lactating dairy cows. *J. Dairy Sci.* **2014**, *97(6)*, 3777–3789. https://doi.org/10.3168/jds.2013-7397.
Staerfl, S.M.; Amelchanka, S.L.; Kälber, T.; Soliva, C.R.; Kreuzer, M.; Zeitz, J.O. Effect of feeding dried high-sugar ryegrass ('AberMagic') on methane and urinary nitrogen emissions of primiparous cows. *Livest. Sci.* **2012**, *150(1–3)*, 293–301. https://doi.org/10.1016/j.livsci.2012.09.019.
Stoldt, A.K.; Derno, M.; Das, G.; Weitzel, J.M.; Wolffram, S.; Metges, C.C. Effects of rutin and buckwheat seeds on energy metabolism and methane production in dairy cows. *J. Dairy Sci.* **2016**, *99(3)*, 2161–2168. https://doi.org/10.3168/jds.2015-10143.
Storlien, T.M.; Prestløkken, E.; Beauchemin, K.A.; McAllister, T.A.; Iwaasa, A.; Harstad, O.M. Supplementation with crushed rapeseed causes reduction of methane emissions from lactating dairy cows on pasture. *Anim. Prod. Sci.* **2017**, *57(1)*, 81–89. https://doi.org/10.1071/AN15287.
van Dorland, H.A.; Wettstein, H.R.; Leuenberger, H.; Kreuzer, M. Effect of supplementation of fresh and ensiled clovers to ryegrass on nitrogen loss and methane emission of dairy cows. *Livest. Sci.* **2007**, *111(1–2)*, 57–69. https://doi.org/10.1016/j.livsci.2006.11.015.
van Vugt, S.J.; Waghorn, G.C.; Clark, D.A.; Woodward, S.L. Impact of monensin on methane production and performance of cows fed forage diets. *Proc. New Zeal. Soc. Anim. Prod.* **2005**, *65*, 362–366. Available online: https://www.nzsap.org/proceedings/2005/impact-monensin-methane-production-and-performance-cows-fed-forage-diets (accessed 18 April 2023).
van Zijderveld, S.M.; Dijkstra, J.; Perdok, H.B.; Newbold, J.R.; Gerrits, W.J.J. Dietary inclusion of diallyl disulfide, yucca powder, calcium fumarate, an extruded linseed product, or medium-chain fatty acids does not affect methane production in lactating dairy cows. *J. Dairy Sci.* **2011**, *94(6)*, 3094–3104. https://doi.org/10.3168/jds.2010-4042.
van Zijderveld, S.M.; Fonken, B.; Dijkstra, J.; Gerrits, W.J.J.; Perdok, H.B.; Fokkink, W.; Newbold, J.R. Effects of a combination of feed additives on methane production, diet digestibility, and animal performance in lactating dairy cows. *J. Dairy Sci.* **2011**, *94(3)*, 1445–1454. https://doi.org/10.3168/jds.2010-3635.
van Zijderveld, S.M.; Gerrits, W.J.J.; Dijkstra, J.; Newbold, J.R.; Hulshof, R.B.A.; Perdok, H.B. Persistency of methane mitigation by dietary nitrate supplementation in dairy cows. *J. Dairy Sci.* **2011**, *94(8)*, 4028–4038. https://doi.org/10.3168/jds.2011-4236.
Warner, D.; Hatew, B.; Podesta, S.C.; Klop, G.; Van Gastelen, S.; Van Laar, H.; Dijkstra, J.; Bannink, A. Effects of nitrogen fertilisation rate and maturity of grass silage on methane emission by lactating dairy cows. *Animal* **2016**, *10(1)*, 34–43. https://doi.org/10.1017/S1751731115001640.
Warner, D.; Podesta, S.C.; Hatew, B.; Klop, G.; van Laar, H.; Bannink, A.; Dijkstra, J. Effect of nitrogen fertilization rate and regrowth interval of grass herbage on methane emission of zero-grazing lactating dairy cows. *J. Dairy Sci.* **2015**, *98(5)*, 3383–3393. https://doi.org/10.3168/jds.2014-9068.
Watt, L.J.; Clark, C.E.F.; Krebs, G.L.; Petzel, C.E.; Nielsen, S.; Utsumi, S.A. Differential rumination, intake, and enteric methane production of dairy cows in a pasture-based automatic milking system. *J. Dairy Sci.* **2015**, *98(10)*, 7248–7263. https://doi.org/10.3168/jds.2015-9463.
Xue, B.; Yan, T.; Ferris, C.F.; Mayne, C.S. Milk production and energy efficiency of Holstein and Jersey-Holstein crossbred dairy cows offered diets containing grass silage. *J. Dairy Sci.* **2011**, *94(3)*, 1455–1464. https://doi.org/10.3168/jds.2010-3663.

Data S1. References used to develop models for predicting the enteric methane emission from lactating dairy cows.

Aguinaga Casañas, M.A.; Rangkasenee, N.; Krattenmacher, N.; Thaller, G.; Metges, C.C.; Kuhla, B. Methyl-coenzyme M reductase A as an indicator to estimate methane production from dairy cows. *J. Dairy Sci.* **2015**, *98(6)*, 4074–4083. https://doi.org/10.3168/jds.2015-9310.

Arndt, C.; Powell, J.M.; Aguerre, M.J.; Wattiaux, M.A. Performance, digestion, nitrogen balance, and emission of manure ammonia, enteric methane, and carbon dioxide in lactating cows fed diets with varying alfalfa silage-to-corn silage ratios. *J. Dairy Sci.* **2015**, *98(1)*, 418–430. https://doi.org/10.3168/jds.2014-8298.

Benchaar, C.; Hassanat, F.; Martineau, R.; Gervais, R. Linseed oil supplementation to dairy cows fed diets based on red clover silage or corn silage: Effects on methane production, rumen fermentation, nutrient digestibility, N balance, and milk production. *J. Dairy Sci.* **2015**, *98(11)*, 7993–8008. https://doi.org/10.3168/jds.2015-9398.

Brask, M.; Lund, P.; Weisbjerg, M.R.; Hellwing, A.L.F.; Poulsen, M.; Larsen, M.K.; Hvelplund, T. Methane production and digestion of different physical forms of rapeseed as fat supplements in dairy cows. *J. Dairy Sci.* **2013**, *96(4)*, 2356–2365. https://doi.org/10.3168/jds.2011-5239.

Ellis, J.L.; Hindrichsen, I.K.; Klop, G.; Kinley, R.D.; Milora, N.; Bannink, A.; Dijkstra, J. Effects of lactic acid bacteria silage inoculation on methane emission and productivity of Holstein Friesian dairy cattle. *J. Dairy Sci.* **2016**, *99(9)*, 7159–7174. https://doi.org/10.3168/jds.2015-10754.

Gidlund, H.; Hetta, M.; Huhtanen, P. Milk production and methane emissions from dairy cows fed a low or high proportion of red clover silage and an incremental level of rapeseed expeller. *Livest. Sci.* **2017**, *197*, 73–81. https://doi.org/10.1016/j.livsci.2017.01.009.

Grainger, C.; Clarke, T.; Beauchemin, K.A.; McGinn, S.M.; Eckard, R.J. Supplementation with whole cottonseed reduces methane emissions and can profitably increase milk production of dairy cows offered a forage and cereal grain diet. *Aust. J. Exp. Agric.* **2008**, *48(2)*, 73–76. https://doi.org/10.1071/EA07224.

Haisan, J.; Sun, Y.; Guan, L.; Beauchemin, K.A.; Iwaasa, A.; Duval, S.; Kindermann, M.; Barreda, D.R.; Oba, M. The effects of feeding 3-nitrooxypropanol at two doses on milk production, rumen fermentation, plasma metabolites, nutrient digestibility, and methane emissions in lactating Holstein cows. *Anim. Prod. Sci.* **2017**, *57(2)*, 282–289. https://doi.org/10.1071/AN15219.

Hamilton, S.W.; DePeters, E.J.; McGarvey, J.A.; Lathrop, J.; Mitloehner, F.M. Greenhouse Gas, Animal Performance, and Bacterial Population Structure Responses to Dietary Monensin Fed to Dairy Cows. *J. Environ. Qual.* **2010**, *39(1)*, 106–114. https://doi.org/10.2134/jeq2009.0035.

Harper, M.T.; Oh, J.; Giallongo, F.; Lopes, J.C.; Roth, G.W.; Hristov, A.N. Using brown midrib 6 dwarf forage sorghum silage and fall-grown oat silage in lactating dairy cow rations. *J. Dairy Sci.* **2017**, *100(7)*, 5250–5265. https://doi.org/10.3168/jds.2017-12552.

Harper, M.T.; Oh, J.; Giallongo, F.; Roth, G.W.; Hristov, A.N. Inclusion of wheat and triticale silage in the diet of lactating dairy cows. *J. Dairy Sci.* **2017**, *100(8)*, 6151–6163. https://doi.org/10.3168/jds.2017-12553.

Hassanat, F.; Gervais, R.; Benchaar, C. Methane production, ruminal fermentation characteristics, nutrient digestibility, nitrogen excretion, and milk production of dairy cows fed conventional or brown midrib corn silage. *J. Dairy Sci.* **2017**, *100(4)*, 2625–2636. https://doi.org/10.3168/jds.2016-11862.

Hassanat, F.; Gervais, R.; Julien, C.; Massé, D.I.; Lettat, A.; Chouinard, P.Y.; Petit, H. V.; Benchaar, C. Replacing alfalfa silage with corn silage in dairy cow diets: Effects on enteric methane production, ruminal fermentation, digestion, N balance, and milk production. *J. Dairy Sci.* **2013**, *96(7)*, 4553–4567. https://doi.org/10.3168/jds.2012-6480.

Hassanat, F.; Gervais, R.; Massé, D.I.; Petit, H. V.; Benchaar, C. Methane production, nutrient digestion, ruminal fermentation, N balance, and milk production of cows fed timothy silage- or alfalfa silage-based diets. *J. Dairy Sci.* **2014**, *97(10)*, 6463–6474. https://doi.org/10.3168/jds.2014-8069.

Hatew, B.; Podesta, S.C.; Van Laar, H.; Pellikaan, W.F.; Ellis, J.L.; Dijkstra, J.; Bannink, A. Effects of dietary starch content and rate of fermentation on methane production in lactating dairy cows. *J. Dairy Sci.* **2015**, *98(1)*, 486–499. https://doi.org/10.3168/jds.2014-8427.

Hollmann, M.; Powers, W.J.; Fogiel, A.C.; Liesman, J.S.; Beede, D.K. Response profiles of enteric methane emissions and lactational performance during habituation to dietary coconut oil in dairy cows. *J. Dairy Sci.* **2013**, *96(3)*, 1769–1781. https://doi.org/10.3168/jds.2012-6039.

Hollmann, M.; Powers, W.J.; Fogiel, A.C.; Liesman, J.S.; Bello, N.M.; Beede, D.K. Enteric methane emissions and lactational performance of Holstein cows fed different concentrations of coconut oil. *J. Dairy Sci.* **2012**, *95(5)*, 2602–2615. https://doi.org/10.3168/jds.2011-4896.

Holter, J.B.; Kim, C.W.; Colovos, N.F. Methionine Hydroxy Analog for Lactating Dairy Cows. *J. Dairy Sci.* **1972**, *55*, 460–465. https://doi.org/10.3168/jds.S0022-0302(72)85516-4.

Hristov, A.N.; Lee, C.; Cassidy, T.; Heyler, K.; Tekippe, J.A.; Varga, G.A.; Corl, B.; Brandt, R.C. Effect of Origanum vulgare L. leaves on rumen fermentation, production, and milk fatty acid composition in lactating dairy cows. *J. Dairy Sci.* **2013**, *96(2)*, 1189–1202. https://doi.org/10.3168/jds.2012-5975.

Hristov, A.N.; Oh, J.; Giallongo, F.; Frederick, T.; Harper, M.T.; Weeks, H.; Branco, A.F.; Price, W.J.; Moate, P.J.; Deighton, M.H.; Williams, S.R.O.; Kindermann, M.; Duval, S. Short communication: Comparison of the GreenFeed system with the sulfur hexafluoride tracer technique for measuring enteric methane emissions from dairy cows. *J. Dairy Sci.* **2016**, *99(7)*, 5461–5465. https://doi.org/10.3168/jds.2016-10897.

Jiao, H.P.; Dale, A.J.; Carson, A.F.; Murray, S.; Gordon, A.W.; Ferris, C.P. Effect of concentrate feed level on methane emissions from grazing dairy cows. *J. Dairy Sci.* **2014**, *97(11)*, 7043–7053. https://doi.org/10.3168/jds.2014-7979.

Livingstone, K.M.; Humphries, D.J.; Kirton, P.; Kliem, K.E.; Givens, D.I.; Reynolds, C.K. Effects of forage type and extruded linseed supplementation on methane production and milk fatty acid composition of lactating dairy cows. *J. Dairy Sci.* **2015**, *98(6)*, 4000–4011. https://doi.org/10.3168/jds.2014-8987.

Lovett, D.K.; Stack, L.J.; Lovell, S.; Callan, J.; Flynn, B.; Hawkins, M.; O'Mara, F.P. Manipulating enteric methane emissions and animal performance of late-lactation dairy cows through concentrate supplementation at pasture. *J. Dairy Sci.* **2005**, *88(8)*, 2836–2842. https://doi.org/10.3168/jds.S0022-0302(05)72964-7.

Lund, P.; Dahl, R.; Yang, H.J.; Hellwing, A.L.F.; Cao, B.B.; Weisbjerg, M.R. The acute effect of addition of nitrate on in vitro and in vivo methane emission in dairy cows. *Anim. Prod. Sci.* **2014**, *54(9)*, 1432–1435. https://doi.org/10.1071/AN14339.

Moate, P.J.; Williams, S.R.O.; Torok, V.A.; Hannah, M.C.; Ribaux, B.E.; Tavendale, M.H.; Eckard, R.J.; Jacobs, J.L.; Auldist, M.J.; Wales, W.J. Grape marc reduces methane emissions when fed to dairy cows. *J. Dairy Sci.* **2014**, *97(8)*, 5073–5087. https://doi.org/10.3168/jds.2013-7588.

Mohammed, R.; McGinn, S.M.; Beauchemin, K.A. Prediction of enteric methane output from milk fatty acid concentrations and rumen fermentation parameters in dairy cows fed sunflower, flax, or canola seeds. *J. Dairy Sci.* **2011**, *94(12)*, 6057–6068. https://doi.org/10.3168/jds.2011-4369.

Muñoz, C.; Hube, S.; Morales, J.M.; Yan, T.; Ungerfeld, E.M. Effects of concentrate supplementation on enteric methane emissions and milk production of grazing dairy cows. *Livest. Sci.* **2015**, *175*, 37–46. https://doi.org/10.1016/j.livsci.2015.02.001.

O'Neill, B.F.; Deighton, M.H.; O'Loughlin, B.M.; Galvin, N.; O'Donovan, M.; Lewis, E. The effects of supplementing grazing dairy cows with partial mixed ration on enteric methane emissions and milk production during mid to late lactation. *J. Dairy Sci.* **2012**, *95(11)*, 6582–6590. https://doi.org/10.3168/jds.2011-5257.

Odongo, N.E.; Bagg, R.; Vessie, G.; Dick, P.; Or-Rashid, M.M.; Hook, S.E.; Gray, J.T.; Kebreab, E.; France, J.; McBride, B.W. Long-term effects of feeding monensin on methane production in lactating dairy cows. *J. Dairy Sci.* **2007**, *90(4)*, 1781–1788. https://doi.org/10.3168/jds.2006-708.

Tekippe, J.A.; Hristov, A.N.; Heyler, K.S.; Cassidy, T.W.; Zheljazkov, V.D.; Ferreira, J.F.S.; Karnati, S.K.; Varga, G.A. Rumen fermentation and production effects of Origanum vulgare L. leaves in lactating dairy cows. *J. Dairy Sci.* **2011**, *94(10)*, 5065–5079. https://doi.org/10.3168/jds.2010-4095.

van Gastelen, S.; Antunes-Fernandes, E.C.; Hettinga, K.A.; Klop, G.; Alferink, S.J.J.; Hendriks, W.H.; Dijkstra, J. Enteric methane production, rumen volatile fatty acid concentrations, and milk fatty acid composition in lactating Holstein-Friesian cows fed grass silage- or corn silage-based diets. *J. Dairy Sci.* **2015**, *98(3)*, 1915–1927. https://doi.org/10.3168/jds.2014-8552.

Vanrobays, M.L.; Bastin, C.; Vandenplas, J.; Hammami, H.; Soyeurt, H.; Vanlierde, A.; Dehareng, F.; Froidmont, E.; Gengler, N. Changes throughout lactation in phenotypic and genetic correlations between methane emissions and milk fatty acid contents predicted from milk mid-infrared spectra. *J. Dairy Sci.* **2016**, *99(9)*, 7247–7260. https://doi.org/10.3168/jds.2015-10646.

Waghorn, G.C.; Clark, H.; Taufa, V.; Cavanagh, A. Monensin controlled-release capsules for methane mitigation in pasture-fed dairy cows. *Aust. J. Exp. Agric.* **2008**, *48(2)*, 65–68. https://doi.org/10.1071/EA07299.

Waghorn, G.C.; Jonker, A.; MacDonald, K.A. Measuring methane from grazing dairy cows using GreenFeed. *Anim. Prod. Sci.* **2016**, *56(3)*, 252–257. https://doi.org/10.1071/AN15491.

Wims, C.M.; Deighton, M.H.; Lewis, E.; O'Loughlin, B.; Delaby, L.; Boland, T.M.; O'Donovan, M. Effect of pregrazing herbage mass on methane production, dry matter intake, and milk production of grazing

dairy cows during the mid-season period. *J. Dairy Sci.* **2010**, *93(10)*, 4976–4985. https://doi.org/10.3168/jds.2010-3245.

Data S2. References used to develop models for predicting the enteric methane emission from lactating dairy cows.

Table S1. External models evaluated for predicting enteric methane emission (EME; g/cow/day) of lactating dairy cows

Models	Equation[1]
Kirchgeßner et al. (1995)	$EME = 10.0 + 4.9 \times MY + 1.5 \times BW^{0.75}$
Yan et al. (2000) Model	$EME = [3.23 + 0.055 \times GEI]/ 0.05565$
Corré (2002)	$EME = [50.0 + 0.01 \times MY \times 365]/365 \times 1000$
Mills et al. (2003) Model I	$EME = [5.93 + 0.92 \times DMI] / 0.05565$
Model II	$EME = [56.27 - (56.27+ 0) \times e^{[-0.028 \times DMI]}]/ 0.05565$
Model III	$EME = [45.98 - (45.98 + 0)\ e^{[-0.003 \times MEI]}]/ 0.05565$
IPCC (2006) Tier II	$EME = [0.065 \times GEI] / 0.05565$
Ellis et al. (2007) Model I	$EME = [3.23 + 0.809 \times DMI] / 0.05565$
Model II	$EME = [3.14 + 2.11 \times NDFI] / 0.05565$
Model III	$EME = [4.08 + 0.068 \times MEI] / 0.05565$
Model IV	$EME = [1.21 + 0.059 \times MEI + 0.093 \times Forage] / 0.05565$
Model V	$EME = [8.56 + 0.139 \times Forage] / 0.05565$
Model VI	$EME = [2.16 + 0.493 \times DMI - 1.36 \times ADFI + 1.97 \times NDFI] / 0.05565$
Model VII	$EME = [5.87 + 2.43 \times ADFI] / 0.05565$
Moate et al. (2011) Model I	$EME = [24.51 - 0.0788 \times EE] \times DMI$
Model II	$EME = [e^{[3.15 - 0.0035 \times EE]}] \times DMI$
Model III	$EME = 2.54 + 19.14 \times DMI$
Nielsen et al. (2013) Model I	$EME = [1.36 \times DMI - 0.125 \times FA - 0.02 \times CP + 0.017 \times NDF] / 0.05565$
Model II	$EME = [1.23 \times DMI - 0.145 \times FA + 0.012 \times NDF] / 0.05565$
Model III	$EME = [1.39 \times DMI - 0.091 \times FA] / 0.05565$
Model IV	$EME = [1.26 \times DMI] / 0.05565$
Model V	$EME = [738 \times DMI\_BW - 0.145 \times FA + 0.013 \times NDF] / 0.05565$
Ramin and Huhtanen (2013) Model I	$EME = [62 + 25 \times DMI] \times 16.0 / 22.4$
Model II	$EME = [20 + 35.8 \times DMI - 0.5 \times DMI^2] \times 16.0 / 22.4$
Storlien et al. (2014) Model I	$EME = [- 1.47 + 1.28 \times DMI] / 0.05565$

Model II	EME = [– 2.76 + 3.74 × NDFI] / 0.05565
Model III	EME = [6.80 + 1.09 × DMI – 0.15 × FA] / 0.05565
Moraes et al. (2014) Model I	EME = [3.247 + 0.043 × GEI] / 0.05565
Model II	EME = [0.225 + 0.042 × GEI + 0.125 × NDF% – 0.329 × EE] / 0.05565
Charmley et al. (2016) Model I	EME = 38.0 + 19.22 × DMI
Model II	EME = [2.14 + 0.058 × GEI] / 0.05565
Santiago–Juarez et al. (2016) Model I	EME = [27.992 + 0.054 × NDF – 0.909 × ME – 0.295 × EE]/ 0.05565
Model II	EME = [29.847 – 0.979 × ME]/ 0.05565
Model III	EME = [3.911 + 0.128 × MY + 1.274 × MP + 2.166 × MF]/ 0.05565
Model IV	EME = [8.967 + 0.141 × MY + 1.626 × MP + 1.919 × MF + 0.054 × NDF – 0.707 × ME]/ 0.05565
Model V	EME = [11.496 + 0.134 × MY + 1.514 × MP + 1.952 × MF – 0.726 × ME]/ 0.05565
Model VI	EME = [–2.483 + 2.132 × MF + 0.069 × NDF – 0.376 × ME– 0.185 × EE + 0.842 × DMI]/ 0.05565
Model VII	EME = [–2.639 + 2.149 × MF + 0.068 × NDF – 0.406 × ME + 0.840 × DMI]/ 0.05565
Model VIII	EME = [–6.123 + 2.342 × MF – 0.573 × MP + 0.072 × NDF – 0.351 × ME – 0.232 × EE + 0.784 × DMI + 0.009 × BW]/ 0.05565
Model IX	EME = [–7.381 + 2.249 × MF + 0.071 × NDF – 0.407 × ME + 0.787 × DMI + 0.009 × BW]/ 0.05565
Model X	EME = [–5.124 + 2.300 × MF + 0.840 × DMI]/ 0.05565
Model XI	EME = [4.544 + 0.773 × DMI]/ 0.05565
Niu et al. (2018) Model	EME = – 0.65 + 12.4 × DMI – 8.78 × EE + 2.10 × NDF% + 16.1 × MF + 0.148 × BW
IPCC (2019) Tier II	EME = [Y_M × GEI] / 0.05565
Eugène et al. (2019) Model	EME = DOMI × MCFe × 0.001
Ribeiro et al. (2020) Model I	EME = [4.15 + 0.822 × DMI]/0.05565
Model II	EME = [3.35 + 0.047 × GEI]/0.05566

[1]MY = milk yield (kg/d); BW = body weight (kg); GEI = gross energy intake (MJ/d); DMI = dry matter intake (kg/d); MEI = metabolizable energy intake (MJ/d); NDFI = neutral detergent fiber intake (kg/d); Forage = forage (% of DM); ADFI = acid detergent fiber intake (kg/d); EE = ether extract (% of DM); FA = fatty acid (g/kg DM); CP = crude protein (% of DM); NDF = neutral detergent fiber (g/kg DM); DMI_BW = dry matter intake relative to BW (g/kg); NDF% = neutral detergent fiber (% of DM); ME = metabolizable energy (MJ/kg DM); MP = milk protein (%); MF = milk fat (%); Y_M = 0.057 if MY > 8500 kg/cow/year, NDF ≤ 35 % DM and DE ≥ 70%, Y_M = 0.06 if MY > 8500 kg/cow/year, NDF ≥ 35 % DM and DE ≥ 70%, Y_M = 0.063 if MY > 8500 kg/cow/year, NDF ≤ 37 % DM and DE ≥ 70%, and Y_M = 0.065 if MY < 5000 kg/cow/year, NDF > 38 % DM and DE ≤ 62%, DOMI = digestible organic matter intake (kg/d); and MCFe = enteric methane conversion factor (expressed in DOMI converted to CH4).

Charmley E.; Williams S.R.O.; Moate P.J.; Hegarty R.S.; Herd R.M.; Oddy V.H.; Reyenga P.; Staunton K.M.; Anderson A.; Hannah M.C. A universal equation to predict methane production of forage-fed cattle in Australia. *Anim. Prod. Sci.* **2016**, *56(3)*, 169–180. https://doi.org/10.1071/AN15365.

Ellis, J.L.; Kebreab, E.; Odongo, N.E.; McBride, B.W.; Okine, E.K.; France, J. Prediction of methane production from dairy and beef cattle. *J. Dairy Sci.* **2007**, *90*(7), 3456–3466. https://doi.org/10.3168/jds.2006-675.

Eugène, M.; Sauvant, D.; Nozière, P.; Viallard, D.; Oueslati, K.; Lherm, M.; Mathias, E.; Doreau, M. A new Tier 3 method to calculate methane emission inventory for ruminants. *J. Environ. Manag.* **2019**, *231*, 982–988. https://doi.org/10.1016/j.jenvm an.2018.10.086.

Intergovernmental Panel on Climate Change (IPCC). 2006 IPCC Guidelines for National Greenhouse Gas Inventories. Global Environmental Strategies (IGES), Japan. Available online: https://www.ipcc.ch/report/2006-ipcc-guidelines-for-national-greenhouse-gas-inventories/ (accessed 20 April 2023).

Intergovernmental Panel on Climate Change - IPCC, 2019. The Refinement to the 2006 Guidelines for National Greenhouse Gas Inventories. Global Environmental Strategies (IGES), Japan. Available onlline: https://www.ipcc.ch/report/2019-refinement-to-the-2006-ipcc-guidelines-for-national-greenhouse-gas-inventories/ (acesses 20 April 2023

Kebreab, E.; Johnson, K.A.; Archibeque, S.L.; Pape, D.; Wirth, T. Model for estimating enteric methane emissions from United States dairy and feedlot cattle. *J. Anim. Sci.* **2008**, *86(10)*, 2738–2748. https://doi.org/10.2527/jas.2008-0960.

Kirchgessner, M.; Windisch, W.; Muller, H.L. Nutritional factors for the quantification of methane production. Ruminant Physiology: Digestion, Metabolism, Growth and Reproduction. Eds. Engelhardt, W. v.; Leonhard-Marek, S.; Breves, G.; Gieseke, D.; Ferdinand Enke Verlag: Stuttgart, 1995; pp. 333–348.

Mills, J. A.; Kebreab, E.; Yates, C.M.; Crompton, L.A.; Cammell, S.B.; Dhanoa, M.S.; Agnew, R.E.; France, J. Alternative approaches to predicting methane emissions from dairy cows. *J. Anim. Sci.* **2003**, *81(12)*, 3141–3150. https://doi.org/10.2527/2003.81123141x.

Moate, P.J.; Williams, S.R.O.; Grainger, C.; Hannah, M.C.; Ponnampalam, E.N.; Eckard, R.J. Influence of cold-pressed canola, brewers grains and hominy meal as dietary supplements suitable for reducing enteric methane emissions from lactating dairy cows. *Anim. Feed Sci. Technol.* **2011**, *166–167*, 254–264. https://doi.org/10.1016/j.anifeedsci.2011.04.069.

Moraes, L.E.; Strathe, A.B.; Fadel, J.G.; Casper, D.P.; Kebreab, E. Prediction of enteric methane emissions from cattle. *Glob. Change Biol.* **2014**, *20(7)*, 2140–2148. https://doi.org/10.1111/gcb.12471.

Moraes, L. E., Strathe, A. B., Fadel, J. G., Casper, D. P., Kebreab, E. (2014). Prediction of enteric methane emissions from cattle. *Global Change Biology*, *20*(7), 2140–2148. https://doi.org/10.1111/gcb.12471.

Nilsen, N.I.; Volden, H.; Åkerlind, M.; Brask, M.; Hellwing, A.L.F.; Storlien, T.; Bertilsson, J. A prediction equation for enteric methane emission from dairy cows for use in NorFor. *Acta Agric. Scand. A Anim. Sci.* **2013**, *63(3)*, 126–130. https://doi.org/10.1080/09064702.2013.851275.

Niu, M.; Kebreab, E.; Hristov, A.N.; Oh, J.; Arndt, C.; Bannink, A.; Bayat, A.R.; Brito, A.F.; Boland, T.; Casper, D.; Crompton, L.A.; Dijkstra, J.; Eugène, M.A.; Garnsworthy, P.C.; Haque, M.N.; Hellwing, A.L.F.; Huhtanen, P.; Kreuzer, M.; Kuhla, B.; Lund, P.; Madsen, J.; Martin, C.; McClelland, S.C.; McGee, M.; Moate, P.J.; Muetzel, S.; Muñoz, C.; O'Kiely, P.; Peiren., N.; Reynolds, C.K.; Schwarm, A.; Shingfield, K.J.; Storlien, T.M.; Weisbjerg, M.R.; Yáñez-Ruiz, D.R.; Yu, Z. Prediction of enteric methane production, yield, and intensity in dairy cattle using an intercontinental database. *Glob. Change Biol.* **2018**, *24(8)*, 3368–3389. https://doi.org/10.1111/gcb.14094.

Ramin, M.; Huhtanen, P. Development of equations for predicting methane emissions from ruminants. *J. Dairy Sci.* **2013**, *96(4)*, 2476–2493. https://doi.org/10.3168/jds.2012-6095.

Ribeiro, R.S.; Rodrigues, J.P.P.; Maurício, R.M.; Borges, A.L.C.C.; Silva, R.R.; Berchielli, T.T.; Filho, S.C.V.; Machado, F.S.; Campos, M.M.; Ferreira, A.L.; Júnior, R.G.; Azevêdo, J.A.G.;

Santos, R.D.; Tomich, T.R.; Pereira, L.G.R. Predicting enteric methane production from cattle in the tropics. *Animal* **2020**, *14(s3)*, s438-s452. https://doi.org/10.1017/S1751731120001743.

Santiago-Juarez, B.; Moraes, L.E.; Appuhamy, J.A.D.R.N.; Pellikaan, W.F.; Casper, D.P.; Tricarico, J.; Kebreab, E. Prediction and evaluation of enteric methane emissions from lactating dairy cows using different levels of covariate information. *Anim. Prod. Sci.* **2016**, *56(3)*, 557–564. https://doi.org/10.1071/AN15496.

Storlien, T.M.; Volden, H.; Almøy, T.; Beauchemin, K.A.; McAllister, T.A.; Harstad, O.M. Prediction of enteric methane production from dairy cows. *Acta Agric. Scand. A Anim. Sci.* **2014**, *64(2)*, 98–109. https://doi.org/10.1080/09064702.2014.959553.

Yan, T.; Agnew, R.; Gordon, F.; Porter, M. Prediction of methane energy output in dairy and beef cattle offered grass silage-based diets. *Livest. Prod. Sci.* **2000**, *64(2–3)*, 253–263. https://doi.org/10.1016/S0301-6226(99)00145-1.

Data S3. Reference of external models evaluated for predicting enteric methane emissions of lactating dairy cows.
